# A Survey of Snakebite Knowledge among Field Forces in China

**DOI:** 10.3390/ijerph14010015

**Published:** 2016-12-26

**Authors:** Chulin Chen, Li Gui, Ting Kan, Shuang Li, Chen Qiu

**Affiliations:** Department of Emergency Nursing, School of Nursing, Second Military Medical University, Shanghai 200433, China; hzla884309@163.com (C.C.); ting.kan@smmu.edu.cn (T.K.); shiny0820@foxmail.com (S.L.); qiuchenqiuying@163.com (C.Q.)

**Keywords:** military personnel, China, snakebite, knowledge, occupational and environmental health

## Abstract

Background: A snakebite is a neglected extrinsic injury associated with high morbidity and global mortality. Members of Chinese field forces are at high risk of snakebites, and their perception and knowledge of snakebites are unknown. The aim of this study is to assess perception and knowledge of snakebites in field forces in southeast China; Methods: A cross-sectional questionnaire-based survey was conducted in July 2016. A total of 216 field force members participated in this study; Results: A total of 10.3% had experienced snakebites and 86.4% rated their demands for knowledge about snakebite as “high”. No significant correlation between the actual and perceived snakebite knowledge status was detected (κ = 0.0237, *p* = 0.3852). Ineffective and harmful traditional first-aid methods, such as the application of tourniquets, sucking the venom out of the wound, and making local incisions, were used by more than three quarters of the respondents. However, pressure immobilization bandages were applied by only 17.3% of members. The proportion of responses for each question was not significantly different among the respondents when considering separate demographic groups; Conclusions: Snakebite knowledge among Chinese field force members is inadequate and in some cases misleading, when focusing on manifestation, prevention, and first-aid. A pragmatic, intensive educational scheme should be undertaken in at-risk populations.

## 1. Introduction

Snakebites are a serious and important medical problem, especially in rural areas of tropical and subtropical developing countries, having been considered by the World Health Organization (WHO) as a neglected extrinsic injury [1,2]. South Asia, southeast Asia, Sub-Saharan Africa, and Latin America, were identified as the most affected regions [2,3]. It is estimated that approximately five million people across the globe are bitten by snakes annually, causing around 125,000 deaths and 400,000 individuals to be permanently disabled or disfigured [4]. However, existing epidemiological data are fragmented and, therefore, the real impact of snakebites is very likely to be underestimated and the full burden of human suffering from snakebites remains unknown [3].

More than 3000 known species of snakes have been identified globally, a vast majority of which have nonvenomous bites (around 60%~80%) [5,6]. However, since snake venoms are the most complex of all natural venoms and poisons, venomous snakebites may cause severe local and systemic symptoms and signs, including tissue damage, generalized myotoxicity, systemic hemorrhage, and acute kidney injury [2,3]. Even a nonvenomous bite has the potential to cause moderately severe damage, resulting from an understandable fear of the bite’s consequences, such as pins and needles of the extremities, vasovagal shock, and being highly agitated and irrational [7,8].

It was reported that most victims were adult males working outdoors, such as farmers, plantation workers, and herdsmen, and the majority of them had little knowledge of snakebites before they were bitten [9,10]. Military personnel assigned to field forces routinely operate under field conditions, and are thereby also vulnerable to snakebites [11,12]. People at high risk of making contact with snakes are much less likely to fall victim to a snakebite if they develop a knowledge of snakes, their habits, and the timely and appropriate first-aid which can be applied to reduce the consequences of snakebites [13].

In China, most of the snakes are distributed in the south and southeast of the country. The Chinese cobra (*Naja naja atra*) belongs to the elapid family (*Elapidae*) with the characteristic “glasses” sign, which is one of China’s top ten poisonous snakes, and is mainly distributed south of the Yangtze River [5]. The Mamushi or Fu-she (*Agkistrodon halys*), is one of the most widely distributed poisonous snakes in China. Other venomous species, such as the Chinese bamboo viper (*Viridovipera stejnegeri*), the Chinese krait (*Bungarus multicinctus*), and the Chinese habu (*Protobothrops mucrosquamatus*), are all common in China. Snakebites and their management in China have been reported to a certain extent [5,14,15,16,17]. However, to the best of our knowledge, this is the first study to investigate knowledge of snakes and snakebites within members of the Chinese military. Our goal is to provide baseline data for understanding the current perception and knowledge of snakebites, and for improving medical education in order to decrease the morbidity and mortality rates of snakebites in China. To achieve this goal, we conducted a survey on the knowledge of snakebites among military personnel in a specific field troop in southeast China.

## 2. Materials and Methods

### 2.1. Design and Sample

The research was formed of a cross-sectional study. A convenience sample was taken from military personnel from a field troop in southeast China. Six platoons that didn’t operate under field conditions at this time were surveyed. A platoon is a military unit containing between 30 and 50 soldiers, and thus 216 military personnel were requested to participate in the study. Those who had engaged in health care were excluded from the study.

A questionnaire (see Supplementary Materials) which could be understood by people with minimal reading ability was developed, based on Guidelines for the Management of Snakebites [18], American Heart Association and American Red Cross Guidelines for First-Aid [19], and the materials used to train medics in China. The questionnaire consisted of three parts: (a) demographic information (six questions), including gender, age, nationality, military service time, educational level, and whether they had relatives or friends working in the healthcare field; (b) self-evaluation (five questions), including self-evaluation about their current perceptions of snakebites and demands for knowledge of snakebites, knowledge acquisition approach, experience of snakebites, and their immediate reaction to the occurrence of a snakebite; (c) knowledge about snakebites (seven questions), including general knowledge about snakebites, prevention, and first-aid for snakebites. The questionnaire comprised multiple-choice questions requiring either single or multiple answers and true or false questions. The scoring used for collecting data on the knowledge of participants was based on the following: (a) true or false questions (every question was worth two and a half points): correct responses were given a score of two and a half and incorrect responses were given a score of zero; (b) multiple-choice questions (every question was worth five points): if a wrong choice was selected, it was counted as zero. If a correct choice was selected, the score of the question increased by one point. A total of five points were given on the condition that all correct choices were selected. Any missing, corrupted, or blank responses, were marked as incorrect. The total score of the questions associated with knowledge ranged from zero to 30 points. The respondents were considered to have a good knowledge of snakebites if they had a score greater than 20 points, an average knowledge if they had a score greater than 10 points and less than or equal to 20 points, and a poor knowledge if they had 10 points or less.

### 2.2. Data Collection

Data were collected in July 2016. The researchers visited a field troop in southeast China. The questionnaire was explained to the military personnel, who then read it and marked their answers on the sheets. It took field force members approximately 15 min to complete the questionnaire, which was given to them in a quiet room, separate from the rest of the field troop. All participants completed the questionnaire.

### 2.3. Statistical Analysis

The data were analyzed using SPSS for Mac version 23.0 program (IBM Corporation, Chicago, IL, USA). Continuous variables were described as means ± standard deviation. Categorical variables were described as counts and percentages. Chi-square analysis was used to analyze statistically significant differences in the responses given among different groups, defined by demographic variables. Agreement between actual and self-evaluated mastery of knowledge status was assessed using weighted Cohen’s Kappa (κ) in SAS (version 9.3, SAS Institute Inc., Cary, NC, USA). Statistical significance was set at *p* value < 0.05.

### 2.4. Ethical Approval

The study was approved by the ethics committee of the Institutional Review Board of the Second Military Medical University and signed informed consent was obtained from each participant (No. CHEC2015-037). The participants were informed of the purpose and procedures of the study. Anonymity and confidentiality were guaranteed.

## 3. Results

Of the 216 subjects recruited for the study, two participants didn’t complete the questionnaire and were excluded from the investigation, resulting in a sample of 214 (99.1%) observations.

### 3.1. Participant Demographics

Demographic characteristics of the respondents are shown in Table 1. All of the participants were male, and 97.2% were below 30 years old. Most of them (95.8%) were of Han nationality. Of the military personnel, 93.1% had served in the army for less than 10 years and approximately 95% had received an education higher than junior high school.

### 3.2. Self-Evaluation

A total of 10.3% (*n* = 22) of individuals reported that they had experienced snakebites, out of which 4.2% (*n* = 9) had been bitten by snakes more than once. The survey revealed that military members generally received information on snakebites from the military medical education (89.7%), and they also used books, magazines, and newspapers (13.5%), television (8.5%), family and friends (8.4%), and Internet (7.5%), in order to access information. In response to self-evaluated current perceptions of snakebites, nearly three fifths of the participants (57.9%, *n* = 124) rated their knowledge as “average”, 35.1% (*n* = 75) rated their knowledge as “good”, and 7.0% (*n* = 15) rated their knowledge as “poor”. With regard to demands for knowledge on snakebites, most of the participants (86.4%, *n* = 185) rated this demand as “high”, whereas only three (1.4%) rated it as “low”, and the others (12.1%, *n* = 26) rated it as “moderate”. It was satisfying to note that upon the occurrence of a snakebite, most of the military personnel (93.9%, *n* = 201) chose to assess the patient’s condition and take action through simple interventions, while 5.6% (*n* = 12) selected to call for help from a military surgeon or medical corpsmen; only one respondent (0.5%) was too nervous to do anything.

### 3.3. Knowledge about Snakebites

Table 2 presents the details of general knowledge that military personnel held when considering snakebites. The proportion of responses within each question was not significantly different among the groups of respondents defined by demography.

Table 3 provides knowledge about prevention and first-aid of military personnel. Similarly, no significant differences were detected in the proportion of responses within each question when considering demographic variables.

### 3.4. Knowledge Misperception

The highest score that military personnel got was 22.0 points, and the lowest was 4.0 points. The average score was 12.04 points ± 3.09 points. Table 4 shows the agreement between actual knowledge status and self-evaluated knowledge status. Weighted Cohen’s Kappa tests revealed no significant relationship between the actual and perceived knowledge status (κ = 0.0237, *p* = 0.3852). A total of 44.4% (*n* = 95) of the respondents accurately assessed their own knowledge status. However, more than a half of the respondents (50.9%, *n* = 109), overestimated their knowledge status and 4.7% (*n* = 10) underestimated their knowledge status.

## 4. Discussion

### 4.1. Self-Evaluation

Military personnel deployed in field operations are always at great risk of contact with snakes [11,12,13]. Although uncommon in our research, 10.3% of the participants had experienced venomous and non-venomous snakebites, and a majority of them considered that they had mastery of the knowledge surrounding snakebites. In addition, a large group of them realized the necessity of gaining knowledge and noted the demand for more information, which implies that there is a need to provide more data relating to snakebites, to those military personnel. Considering the circumstances of snakebites, the distance from bite location to hospitals may delay the timely administration of anti-venom, the most rational and effective treatment [20], so appropriate first-aid treatments are warranted to reduce mortality and morbidity [21]. To our satisfaction, almost every participant in the survey chose to give first-aid immediately following a snakebite.

Our results showed that the snakebite knowledge of field force members mainly arose from military medical education, indicating that a well-designed and comprehensive medical training program, combined with updating international guidelines, should be implemented in order to convey the appropriate messages. Moreover, televisions, the Internet, and books, newspapers, and magazines, are all feasible methods for field force members to acquire information, and thus making full use of these media outlets contributes to a better understanding of snakebites. To our knowledge, the military medical education is usually given by medics, the most prominent source of the platoon’s healthcare knowledge. A lack of uniform and updated training materials distributed to the military personnel is probably the reason why there exists a gap in their knowledge of snakebites.

Despite more than one third (35%, *n* = 75) of the participants stating that they had a “good” knowledge of snakebites in the present study, only one of them was officially classified as “good” (0.5%), whereas the others were “average” (72.9%) or “poor” (26.6%), according to our score-based classification. The average score only stood at 12.04 points ± 3.09 points, which indicated that the snakebite knowledge of the participants was inadequate. Besides, only 44.4% (*n* = 95) of the respondents accurately assessed their own knowledge status, and more than half of the respondents (50.9%, *n* = 109) overestimated their knowledge status. The high prevalence of misperceptions of snakebite knowledge in field force members is alarming, because their perception could affect their preventive measures and first-aid. They may have great confidence in applying the appropriate first-aid on occurrence of the snakebite, but inversely, their measures may delay medical treatment or cause further harm [18]. This finding reinforces the significance of recognizing the misunderstanding of snakebite knowledge in field forces and conveying correct knowledge to those military personnel.

### 4.2. General Knowledge about Snakebites

General knowledge about snakebites within field force groups in this survey was not as satisfactory as expected. The rate of correct answers for “high-incidence period” was below 40%. Most of the field force members knew that snakebites occurred frequently during nighttime and summer, but were unaware that a number of snakebite cases occurred after the rain. Rain may wash debris and snakes into gutters at the edge of roads, hence, when walking after heavy rains, especially after dark, people should be careful [18].

Early recognition of the manifestation of a snakebite is essential for targeted first-aid treatment. Symptoms and signs vary according to the species of the snake. Local manifestations, such as bleeding and swelling, severe pain, and systemic manifestations such as nausea and vomiting, drowsiness and weakness, and dizziness, are all common signs and symptoms of a snakebite [18]. However, our study suggested that only around 30% of the participants were able to correctly identify all of these manifestations, indicating that their knowledge surrounding snakebite manifestations was fragmentary.

Although there is no simple rule for identifying a venomous snake, since some harmless snakes have evolved to appear almost identical to venomous ones, some notorious venomous snakes can be recognized by their shape, size, pattern of markings, and behaviors [18]. Determining whether the biting snake is a venomous species can make a difference when considering simple superficial wound care and expensive access to hospital care [7]. In our investigation, nearly 70% of the respondents could not identify the venomous snakes by the oval shaped head and regular teeth marks. We decided that the result arose from either their decision that the depicted characteristics of venomous snakes were incorrect, or their belief that it was unreasonable to identify the venomous snakes by their characteristics. Therefore, further research could be designed to explore the exact reason for this response and further information about snakes could be conveyed in order to eliminate their misunderstanding.

The Centers for Disease Control and Prevention (CDC) recommends that people do not pick up a snake or try to trap it, because this carries the risk of a snakebite [22]. It was gratifying that a vast majority of field force members were aware that even an accidental scratch from the fang of a snakes’ severed head may inject venom into their body.

Accordingly, an educational program which teaches high-incidence periods of snakebites and possible signs and symptoms of various species of snakes, is recommended to enhance snakebite knowledge among field force members.

### 4.3. Knowledge about Preventive Measures and First-Aid

From a human health perspective, it is vitally important to understand snakebite prevention among people who are subject to snakebites [23], as the best treatment for snakebites is prevention [24]. Preventive measures are recommended in snakebite prone regions [25]. In our study, preventive measures such as wearing long pants and boots, especially when walking in undergrowth or in the dark, and never resting near the holes, nests, and other hidden places where snakes might rest, were well understood by the respondents. However, only one-half of the respondents were conscious of using a light when walking at night. What’s worse, more than three fifths of the participants chose to walk straight over, rather than step on, facing rocks or logs. Snakes may be sunning themselves on the side of rocks or logs [18], and thus, stepping on the rocks or logs could decrease the risk of being bitten.

Appropriate first-aid performed by the victim himself/herself, or by others who are present and able, is advocated by WHO [18], the American Heart Association and the American Red Cross [19]. First-aid aims to retard systemic absorption of venom, control dangerous and distressing early symptoms of envenoming, prevent complications, and preserve life, before victims receive medical care. The practice of inappropriate first-aid is identified as one of the major challenges of ensuring appropriate care [26]. Unfortunately, our results showed that only 2.3% of respondents were able to correctly answer the first question on first-aid, and only 0.9% were able to correctly identify the second, which is far from satisfactory. When considering how to deal with the snakebite wound, a very high percentage of the respondents attempted to suck the venom out of the wound and make local incisions at the site of the bite, both of which are proved to be ineffective and even dangerous first-aid techniques [19]. Similar useless and harmful methods, such as the use of ice packs and massaging the bite wound, were also selected by a small group of the respondents. Vigorous cleaning should be avoided as this may increase absorption of the venom and local bleeding, according to WHO guidelines. However, a shortage of anti-snake venom and poor access to appropriate anti-venoms exist worldwide, especially in rural areas in developing countries [27,28]. Considering these factors, Chinese experts agree that it is acceptable to gently rinse the wound with water as soon as possible, in order to remove some of the venom following a snakebite. Therefore, it was understandable that it was undertaken by 87.9% of the respondents in our study.

Reassuring the snakebite victim is recommended, as they may be very anxious [18] and this form of first-aid had been adopted by more than three quarters of the respondents. A total of 73.8% of the investigated military personnel were aware of immobilizing the whole of the victim’s body, specifically the bitten limb, which is a desirable practice for decreasing venom absorption [29]. Application of tourniquet is a dangerous intervention, carrying a high risk of well-known adverse consequences, such as ischemic damage and rhabdomyolysis, contributing to amputation and skin grafting [5,29,30,31]. However, a substantial proportion of the respondents (93.5%) said that applying a tourniquet was a good idea. This proportion is much higher than previous studies conducted in Sri Lanka [29], but is consistent with those pursued in Bangladesh [32]. Consequently, there is an urgent need to avoid inappropriate traditional treatments, including application of chemicals, herbs or ice packs, and use of (black) snake stones, which may delay presentation, distort the clinical picture, and even cause infection, gangrene, and other complications [2,18]. Moreover, only a small number of the participants decided to apply a pressure immobilization bandage, a safe way to delay toxicity by slowing lymph flow, unless a neurotoxic elapid can be excluded [2,33,34]. We consider that the unawareness of applying a pressure immobilization bandage results from a lack of confidence and a poor retention of the skill of pressure immobilization. It has been demonstrated that inadequate pressure is ineffective and too much pressure may cause local tissue damage, and once learned, retention of the skill of proper pressure and immobilization application is poor [19]. Accordingly, it is a challenge to find an effective way to teach the application of the correct snugness of the bandage, and we need to make it possible for military personnel to receive proper first-aid more quickly.

Above all, we suggest that the lack of applying WHO recommended first-aid, associated with an inclination of field force members to use incisions, tourniquets, and suck out the venom, offers an opportunity for military educational intervention.

### 4.4 Limitations

There are several limitations in our research. Firstly, since no relevant data was available to calculate the sample size, the convenience sample and the small sample size devalue the representativeness of the sample. Therefore, a generalization of the study findings is restricted. Secondly, the questions included in the questionnaire relating to the knowledge of snakebites are limited and were developed without fully knowing the detailed contents of materials used to train military personnel, which may not fully reflect current status of the knowledge of participants. Thirdly, recommendations and survey questions are based on snakebite risk in the region of the survey, so they may not be universally applicable.

## 5. Conclusions

Snakebites often occur in Chinese field forces. Our research revealed that snakebite knowledge within these field forces was inadequate and in some cases misleading when considering manifestation, prevention, and first-aid. Military personnel desired more information on snakebites. In order to address these issues, we suggest that a pragmatic, intensive educational effort should be focused on basic knowledge of snakes, prevention, and first-aid measures undertaken in these at-risk populations.

## Figures and Tables

**Table 1:** Demographic characteristics of the respondents (*n* = 214).

Characteristics	*n* (%)
Gender	
Male	214 (100.0)
Female	0 (0.0)
Age (years)	
≤20	58 (27.1)
21~30	150 (70.1)
31~40	5 (2.3)
No response	1 (0.5)
Nationality	
Han	205 (95.8)
Minorities	9 (4.2)
Military Service Time (years)	
≤5	156 (73.9)
6~10	41 (19.2)
11~15	14 (6.5)
No response	3 (1.4)
Educational level	
Bachelor degree or above	49 (22.9)
College degree	40 (18.7)
Vocational degree	42 (19.6)
Senior high school diploma	69 (32.2)
Junior high school diploma or below	12 (5.6)
No response	2 (0.9)
Relatives or friends working in healthcare field	
Yes	87 (40.7)
No	127 (59.3)

**Table 2:** General knowledge about snakebite (*n* = 214).

Questions	*n* (%) **
Which of the following are high-incidence periods of snakebite?	
① Noontime	10 (4.7)
② Nighttime *	164 (76.6)
③ After the rain *	101 (47.2)
④ Summer *	206 (96.3)
⑤ Winter	1 (0.5)
The correct answers: ②③④	76 (35.5)
What are the symptoms of snakebite?	
① Local bleeding and swelling *	166 (77.6)
② Severe pain at the site of the bite *	140 (65.4)
③ Nausea and vomiting *	137 (64.0)
④ Drowsiness and weakness *	128 (59.8)
⑤ Dizziness *	175 (81.8)
No response	3 (1.4)
The correct answers: ①②③④⑤	68 (31.8)
When handling dead snakes, people may suffer venom injection by an accidental scratch from the fang of a snake’s severed head.	
True *	194 (90.7)
False	20 (9.3)
The venomous snake’s head is usually oval shaped, with regular teeth marks	
True	66 (30.8)
False *	146 (68.2)

* The correct answer; ** The first number listed represents the number of responses and the number in parentheses represents the percentage number of responses.

**Table 3:** Knowledge about prevention and first-aid (*n* = 214).

Questions	*n* (%) **
Which of the following behaviors are likely to cause a snakebite during field training?	
① Wear proper shoes or boots and long trousers instead of sandals or bare-foot.	22 (10.3)
② Straight over rocks or logs rather than step on them. *	64 (29.9)
③ Do not use a light (torch, flashlight or lamp) when walking at night. *	113 (52.8)
④ Rest near the holes, nests and other hidden places. *	184 (86.0)
No response	3 (1.4)
The correct answers: ②③④	16 (7.5)
What would you do with the wound if someone suffered a snakebite?	
① Rinsing (not scrubbing) the wound with water as soon as possible. *	188 (87.9)
② Attempt to suck the venom out of the wound.	161 (75.2)
③ Application of ice packs.	60 (28.0)
④ Making local incisions at the site of the bite.	189 (88.3)
⑤ Application of alcohol.	57 (26.6)
⑥ Massage the bite wound.	10 (4.7)
The correct answers: ①	5 (2.3)
Apart from calling for help, which of the following first-aid measures would you take if someone suffered a snakebite?	
① Tell him/her to stay calm. *	166 (77.6)
② Immobilize the victim’s whole body, especially the wounded limb. *	158 (73.8)
③ Raise the site of the bite above the level of the person’s heart.	28 (13.1)
④ Application of tight tourniquets around the upper part of the limb.	200 (93.5)
⑤ Applying a pressure immobilization bandage. *	37 (17.3)
No response	1 (0.5)
The correct answers: ①②⑤	2 (0.9)

* The correct answer; ** The first number listed represents the number of responses and the number in parentheses represents the percentage number of responses.

**Table 4:** The agreement between actual knowledge status and self-evaluated knowledge status (*n* = 214).

Actual Knowledge Status	Self-Evaluated Knowledge Status, *n* (%)				Weighted Kappa, κ
	Poor	Average	Good	Total	
Poor	6 (2.8)	34 (15.9)	17 (7.9)	57 (26.6)	0.0237
Average	9 (4.2)	89 (41.6)	58 (27.1)	156 (72.9)	
Good	0 (0.0)	1 (0.5)	0 (0.0)	1 (0.5)	
Total	15 (7.0)	124 (57.9)	75 (35.0)	214 (100.0)	

## Supplementary Materials

The following are available online at www.mdpi.com/1660-4601/14/1/15/s1, Table S1: Demographic information, Table S2: Self-evaluation, Table S3: Knowledge about snakebite.
