# Supplementary material for: A Survey of Snakebite Knowledge among Field Forces in China
Source: Int J Environ Res Public Health. 2016 Dec 26;14(1):15. doi: 10.3390/ijerph14010015 (PMC5295266; doi:10.3390/ijerph14010015)
Supplement: Supplementary file 1 [file ijerph-14-00015-s001.pdf]

Supplementary Materials: A Survey of Snakebite Knowledge among Field Forces in China

Chulin Chen, Li Gui, Ting Kan, Shuang Li and Chen Qiu

Snakebite Knowledge Questionnaire

We are conducting a survey regarding your knowledge about snakebite. We ask if you could please spend 15 min to help fill out this questionnaire. The information you provide will be only used for academic research and is completely confidential. Please choose the truthful answers based on your own condition. Thank you for your participation.

Table S1. Demographic information.

No.	Questions	Options/Answers
A.1.	Gender	□ Male<br>□ Female
A.2.	Age	□
A.3.	Nationality	□
A.4.	How many years have you been on duty?	□
A.5.	What's the highest education level you have obtained?	□ Bachelor's degree or above<br>□ College degree<br>□ Vocational degree<br>□ High school diploma<br>□ Junior high school diploma or below
A.6.	Are there any medical staff in your family, relatives, and friends?	□ Yes<br>□ No

Table S2. Self-evaluation.

No.	Questions	Options/Answers
B.1.	How would you rate your knowledge about snakebite?	□ 3-Good<br>□ 2-Average<br>□ 1-Poor
B.2.	How would you rate your demands for knowledge about snakebite?	□ 3-High<br>□ 2-Moderate<br>□ 1-Low
B.3.	Where did you obtain the knowledge about snakebite?	□ Military medical education<br>□ Television<br>□ Books/Magazines/Newspapers<br>□ Internet<br>□ Families/Friends<br>□ Others:
B.4.	Have you ever experienced snakebite?	□ Yes (Number of times: _____)<br>□ No
B.5.	What was your first reaction when you or your companions suffered snakebite?	□ Too nervous to do anything<br>□ Call for surgeon or medical corpsmen<br>□ Take simple interventions immediately

Table S3. Knowledge about snakebite.

No.	Questions	Options/Answers
C.1.	Which of the following are high-incidence periods of snakebite?	□ Noontime
		□ Nighttime
		□ After the rain
		□ Summer
		□ Winter
C.2.	What are the symptoms of snakebite?	□ Local bleeding and swelling
		□ Severe pain at the site of the bite
		□ Nausea and vomiting
		□ Drowsiness and weakness
		□ Dizziness
C.3.	When handling dead snakes, people may suffer venom injection by an accidental scratch from the fang of a snake’s severed head.	□ True
		□ False
C.4.	The venomous snake’s head is usually oval shaped, with regular teeth marks.	□ True
		□ False
C.5.	Which of the following behaviors are likely to cause a snakebite during field training?	□ Wear proper shoes or boots and long trousers instead of sandals or bare-foot
		□ Straight over rocks or logs rather than step on them
		□ Do not use a light (torch, flashlight or lamp) when walking at night
		□ Rest near the holes, nests and other hidden places
C.6.	What would you do with the wound if someone suffered a snakebite?	□ Rinsing (not scrubbing) the wound with water as soon as possible
		□ Attempt to suck the venom out of the wound
		□ Application of ice packs
		□ Making local incisions at the site of the bite
		□ Application of alcohol
		□ Massage the bite wound
C.7.	Apart from calling for help, which of the following first-aid measures would you take if someone suffered a snakebite?	□ Tell him/her to stay calm
		□ Immobilize the victim's whole body, especially the wounded limb
		□ Raise the site of the bite above the level of the person's heart
		□ Application of tight tourniquets around the upper part of the limb
		□ Applying a pressure immobilization bandage

© 2016 by the authors; licensee MDPI, Basel, Switzerland. This article is an open access article distributed under the terms and conditions of the Creative Commons by Attribution (CC-BY) license (http://creativecommons.org/licenses/by/4.0/).
